# Victimization experiences, internalizing problems and family bonds among adolescents from the UK: multi-group structural equation modeling using an intersectionality-informed approach

**DOI:** 10.1038/s41598-024-80342-0

**Published:** 2024-11-20

**Authors:** Susanne Birnkammer, Cara L. Booker, Claudia Calvano

**Affiliations:** 1 Clinical Child and Adolescent Psychology and Psychotherapy, Department of Education and Psychology, Freie Universität Berlin, 14195 Berlin, Germany; 2 Institute for Social and Economic Research, University of Essex, Colchester, UK; 3 German Center for Mental Health (DZPG), partner site Berlin-Potsdam, Berlin, Germany

**Keywords:** Psychology, Public health

## Abstract

Adolescents from marginalized backgrounds are at increased risk for victimization experiences, which was shown for ethnic minorities and females. However, an intersectional approach has rarely been taken in research. Using the Understanding Society Youth Panel, multigroup structural equation models were conducted to identify cross-group variation among adolescents aged 10–15 years in the relation between victimization experiences (verbal, physical and cyberbullying, discrimination) and internalizing problems (loneliness, emotional problems, life satisfaction), along with a mediating effect of family bonds (family support, parental communication). The groups white male (*n* = 280), white female (*n* = 280), Black, Asian and minority ethnic (BAME) male (*n* = 219) and BAME female (*n* = 279) were included in the analyses. Across all groups, victimization experiences were negatively associated with family bonds. Only among BAME male adolescents, experiences of victimization were related to more internalizing problems. No mediating effects of family bonds on the relation between victimization and internalizing problems were identified in any group. The results support an intersectionality-informed approach and the necessity of implementing preventative anti-bullying and family strengthening interventions among adolescents.

## Introduction

Children and adolescents are increasingly exposed to various forms of victimization, which can encompass several forms of harm, ranging from overt acts of bullying to insidious forms of discrimination. Bullying, a repeated and repetitive act of verbal, physical or digital harassment of an individual occurring in an unbalanced position of power^1^ reached global prevalence rates for childhood and adolescence from 19 to 60% for the last 12 months^2–4^. Studies point towards an overlap between face-to-face and cyberbullying^5,6^, with prevalence rates from 7 to 47% of children and adolescents being exposed to both^2,7,8^.

Beyond bullying, discrimination is another critical form of victimization that many young people face. Discrimination involves disadvantaging an individual due to the attribution of artificially created social group constructs like race or gender^9^ with notable prevalence rates ranging from 22 to 25% for racism among BAME (Black, Asian and minority ethnic) adolescents^10^ to 51% for sexism for female adolescents^11^. Moreover, BAME adolescents^12^ or first generation immigrant adolescents^13^ are exposed to experiences of vicarious^14^ and self-directed victimization and violence experiences^15^, such as discrimination^16^, physical^17^ and verbal bullying^18^ or an overlap in the form of polyvictimization^19^. While these forms of victimization affect both male and female adolescents, female adolescents face the additional threat of sexualized victimization^20^.

Therefore, to accurately depict the realities faced by marginalized adolescents, it is essential to consider intersectional frameworks^21^. Intersectionality integrates the interdependence of various characteristics of discrimination^11,22^ examining individuals in terms of multiple social group affiliations like gender and race. Originally coined by Kimberlé Crenshaw^21^, intersectionality emphasizes that oppression is not experienced in isolation; rather, the dynamic interaction of multiple identities—such as race, gender, class, and sexuality—creates unique and intensified experiences of discrimination and disadvantage. According to empirical findings on these intersections, belonging to several marginalized groups increases the risk of being affected by victimization^23,24^. For instance, in a study among *n *= 1,957 adolescents from the USA, BAME female adolescents reported more vicarious experiences of threatening or violent encounters than their white peers^25^. Similarly, among *n *= 6,750 immigrant and US-born youth, white American girls were less likely to experience school victimization than white male youth, but Asian American immigrant girls were 40.9% more likely to be victimized at school than white male youth^26^. These findings underscore the critical role of intersectionality in the context of victimization. The overlapping social identities of race and gender do not merely aggregate risks but rather interact synergistically, resulting in compounded vulnerabilities that may elevate both the severity and frequency of victimization.

Although bullying and discrimination experiences can be considered as differentiable forms of victimization^27^, these constructs tend to co-occur, especially among marginalized individuals, which may reinforce negative effects on mental health^27^. A common consequence of victimization is the development of internalizing problems^28–30^. These impacts include depressive symptoms^31–33^, suicidal tendencies^34,35^, anxious feelings^31,36^, feelings of loneliness^37,38^ and lower life satisfaction^39,40^. This reflects the wide-ranging impact of victimization on mental health. Recent research has highlighted that life satisfaction is a general measure of well-being and a critical transdiagnostic marker of mental health, reflecting how individuals perceive and emotionally respond to their life circumstances. This perspective aligns with findings from the recent study by Jovanović (2022)^40^, which emphasizes that life satisfaction is a significant component of internalizing behaviors due to its integrative role in cognitive and affective processes.

Recognizing the broad and severe consequences of victimization experiences on adolescent mental health, research has focused on the identification of protective factors. Positive family relationships have been identified as potential protective factors against the adverse effects of victimization experiences on mental health^41,42^. During this period of heightened vulnerability, strong family bonds—marked by adequate support^43^ and effective communication^44^—play a crucial role in mitigating adolescents’ internalizing problems. Moreover, family communication may buffer the relationship between victimization experiences and internalizing problems^45^. Specifically, factors such as intrafamilial contact through joint family dinners^46^ and quality of father-child communication^47^ were associated with reduced levels of internalizing problems that are linked to victimization experiences among children and adolescents.

However, the examination of the multifaceted dynamics of family bonds as protective factors against victimization experiences and their subsequent impact on adolescents’ mental health lacks the consideration of the nuanced intersectional influences of social groups based on race and gender. While aspects of positive family bonds like family cohesion^48^, supportive communication^49^ and active listening^50^ were found as potential buffering factors affecting the impact of bullying on internalizing symptoms among Black and Multiracial African-American adolescents, studies on gender effects showed inconsistent findings, including higher buffering effects of family factors in either female^51^ or male^52^ adolescents. Up to now, studies primarily focused on single discrimination categories and overlooked the intersection of gender and race for the relationships between victimization experiences, internalizing problems and family bonds. The present study aims to bridge this gap and examine the following questions using an intersectional perspective, considering four groups based on the intersection of gender (female vs. male) and race (BAME vs. White): (1) How do the groups differ in the association between victimization experience and internalizing problems? and (2) How does the potentially buffering influence of family bonds vary between the groups in terms of victimization experiences and internalizing problems?

We expect a positive relation between victimization experiences and internalizing problems and that this connection is stronger for the group at higher risk of intersectional discrimination, i.e. BAME female adolescents, compared to the other three groups. Concerning the second research question, we hypothesize that among BAME adolescents, a greater mitigating effect of family bonds regarding the influence of victimization experiences on internalizing problems will be identified compared to white participants. Since the intersection of gender and race has not yet been investigated regarding the mediating role of family bond, no hypothesis on the influence of gender in intersection with race is made and therefore, this research question is exploratively investigated.

## Methods

### Participants

Data was drawn from the panel study Understanding Society: the UK Household Longitudinal Study (UKHLS)^53^, by using the data from the youth questionnaire for 10–15-year-olds adolescents collected in wave 13, covering the years 2021/2022. Participants were young people aged 10–15 whose parents or responsible adults consented to their participation in the 13th wave of UKHLS. Included in the analyses were only participants who lived with at least one biological parent (either their biological mother or father) to ensure the applicability of the family-related items. Exclusion criterion was no parental or young person informed consent as well as no participation of at least one adult household member in the survey. The questionnaire for the adolescents was posted to the households and/or a parent with a £10 voucher for the respective adolescent and a pre-stamped return envelope.

### Data materials

*Demographics* With the exception of the net household income variable, which was taken from the parents’ questionnaire, the demographic variables age, gender and ethnicity were measured as self-reports using the pen and pencil questionnaire. Age was determined by stating the date of birth, and gender could be selected from two answer options female and male. Information on participants’ ethnicity derived from the previous wave’s questionnaire and was based on a self-assessment of ethnic group affiliation “*Which of the following groups do you think you belong to*?”. The participants could select between the following categories: White (British, English, Scottish, Welsh, Northern Irish, Irish, Gypsy or Irish Traveller, Any other White background), Asian or British Asian (Indian, Pakistani, Bangladeshi, Chinese, Any other Asian background), Black/African/Caribbean or Black British (Caribbean, African, Any other Black background), Mixed (White and Black Caribbean, White and Black African, White and Asian, Any other Mixed background) and Other (Arab, Any other ethnic group). In alignment with the self-designation term BAME, which serves as a collective identification for several ethnic groups in the UK, the ethnic group affiliations were subsequently categorized into two groups: White and BAME (comprising Black, Asian, and Other Minority Ethnic groups). To reflect the complexity and intersectionality inherent in the BAME classification framework, individuals from the ‘Mixed’ ethnic group were also included within the BAME category. Participants were therefore grouped into the BAME category if their responses were not classified as white.

*Victimization Experiences* Victimization experiences were assessed by (1) the report on discrimination experiences over the past year, assessed by selecting the applicable experiences out of a list of eight different discrimination categories and (2) by the frequency of verbal, physical, and cyberbullying over the past six months. Discrimination experiences were assessed using the following item*: “In the last 12 months, have you been treated differently by others, in a negative way, for any of these reasons?”*. Multiple choice was permitted, allowing children and adolescents to select from categories including gender, origin/ethnicity, age, religion, health/disability, body physics, language/accent, or other. For the analyses, a single variable was generated representing the number of discrimination categories experienced in the last 12 months, with 0 = no discrimination in the last 12 months to the highest possible value being 8 = discriminated on the basis of eight characteristics in the last 12 months. Bullying experiences were assessed with the following questions: *“How often do you get bullied in other ways at school such as getting called names, getting left out of games, or having nasty stories spread about you on purpose?”*, *“How often do you get physically bullied at school, for example getting hit, pushed around or threatened, or having belongings stolen?”*, and *“How often do you get bullied online, such as getting called hurtful names, having nasty stories spread about you, being bothered or threatened?”*. These questions assessed bullying frequency over the last six months with response options ranging from 1 = *“Never”* to 4 = *“A lot (a few times every week)”*.

*Family bonds* The latent variable family bonds was quantified using three manifest variables from the questionnaire. This includes perceived family support with the item “*Do you feel supported by your family, that is the people who live with you?*” (response categories 1 = I feel supported by my family in most or all of the things I do, 2 = I feel supported by my family in some of the things I do, 3 = I do not feel supported by my family in the things I do), and the communication frequency of significant topics with the mother and with the father (“*How often do you talk to your mother/father, about things that matter to you?*”; response categories 1 = “Most days” till 5 = “Don’t have a mother/father”). In order to facilitate interpretation, the response options for the variables were recoded, meaning a high value indicated a higher family bond.

*Internalizing Problems* The latent variable internalization problems was measured using three observed variables: Loneliness, emotional problems and life satisfaction. Loneliness was assessed by one item “*How often do you feel lonely*” (answer options: 1 = “Hardly ever or never”, 2 = “Some of the time”, 3 = “All of the time”). Emotional problems comprised the subscale of the Strengths Difficulty Questionnaire^54^ for 11–17 year olds, which is calculated using the mean value of 5 items on bodily complaints (“*I often get headaches, stomach aches or sickness*”), worries (“*I worry a lot*”), an unhappy mood (“*I am often unhappy, down-hearted or tearful*”), nervousness (“*I am nervous in new situations, I easily lose confidence*”) and anxiety (“*I have many fears, I am easily scared*”). Response options were 0 = “not true”, 1 = “somewhat true” and 2 = “certainly true”. Life satisfaction was assessed through one item “*Which smiley best describes how you feel about your life as a whole?*”, to which the participants could respond using 7 different smileys, showing 1 = “completely happy” and with a smiling face and 7 = “not at all happy” and with a sad face. Thus, higher values correspond to reduced life satisfaction.

### Ethics

The present study was conducted as a secondary data analysis within the framework of the longitudinal data collection of the Understanding Society surveys, all methods described have been approved by the University of Essex Ethics Committee. Approvement included asking consent for all data linkages except to health records. Requesting consent for health record linkage was approved at Wave 1 by the National Research Ethics Service (NRES) Oxfordshire REC A (08/H0604/124), at BHPS Wave 18 by the NRES Royal Free Hospital and Medical School (08/H0720/60) and at Wave 4 by NRES Southampton REC A (11/SC/0274). All methods were performed and reported in accordance with the guidelines of the University of Essex Ethics Committee and the Declaration of Helsinki for studies involving human subjects. Informed consent was obtained from the parents or legal guardians for the adolescents’ participation in the study.

### Statistical analysis

Data cleaning, descriptive analyses, pairwise group comparison and missing values tests were performed using the software IBM SPSS 29.0.0.0 for Windows devices. Descriptively, the mean values in victimization experiences, internalizing problems and family bonds were compared across all four groups using the robust Kruskal-Wallis test, with Bonferroni corrected two-tailed p-values. Internal consistency of the latent variables was determined using the McDonalds Omega^55^, a robust indicator for normal distribution violations and correlated errors^56^.

Regarding the research questions and hypothesized mediational model, we first conducted a confirmatory factor analysis for specification of the SEM. The CFA and SEM were both conducted using the lavaan package^57^ within the R statistical environment (version 4.3.2). Missing data frequencies ranged from 0.3 to 2.8% and were tested for its randomness using the Little MCAR^58^ test with significant *Chi*^*2*^(1583) = 1860.35, *p *< 0.001. As missing at random (MAR) or not missing at random (NMAR) NMAR were assumed, relevant model indicators were imputed using multiple imputation with an expectation-maximization algorithm (EM) of 25 iterations. Within this sample, a group size difference of a maximal ratio of 2.92 existed, potentially masking certain effects such as factorial invariance^59^, hence, a subsampling approach was applied. Using the SPSS software program, 280 cases were randomly selected for the white male and female group. Multivariate normal distributions of the indicator variables were tested using the Mardia-Kurtosis test^60^ and subsequently rejected (Mardia Skewness = 7626.09 (*p *< 0.001) and Mardia Kurtosis = 79.67 (*p *< 0.001)), therefore, the estimates were calculated with the maximum likelihood method (MLM) and a robust Satorra-Bentler estimation approach^61^.

A multigroup CFA was conducted to test the latent variables “victimization experience”, “family bonds” and “internalizing problems” within the model structure for the social groups. The variances of the latent variables in the CFA model were fixed at 1. For this purpose, model fit statistics and factor loadings for the latent variables were examined using Chi^2^ statistics, the model was successively modified based on model indices. Based on Hu and Bentler (1999), benchmark values for the global fit of a model include CFI close to 0.95, a cut-off of up to 0.06 for RMSEA and 0.08 for SRMR^62^. To enable group comparisons between the social groups within the SEM, the configural and metric measurement invariance was examined with a cut-off value for an assumed measurement invariance of ∆CFI = 0.01^63^. For testing measurement invariance between groups, the multi-group configural model was then estimated and compared with a constrained model having equal factor loadings among the four social groups.

**Fig. 1:**
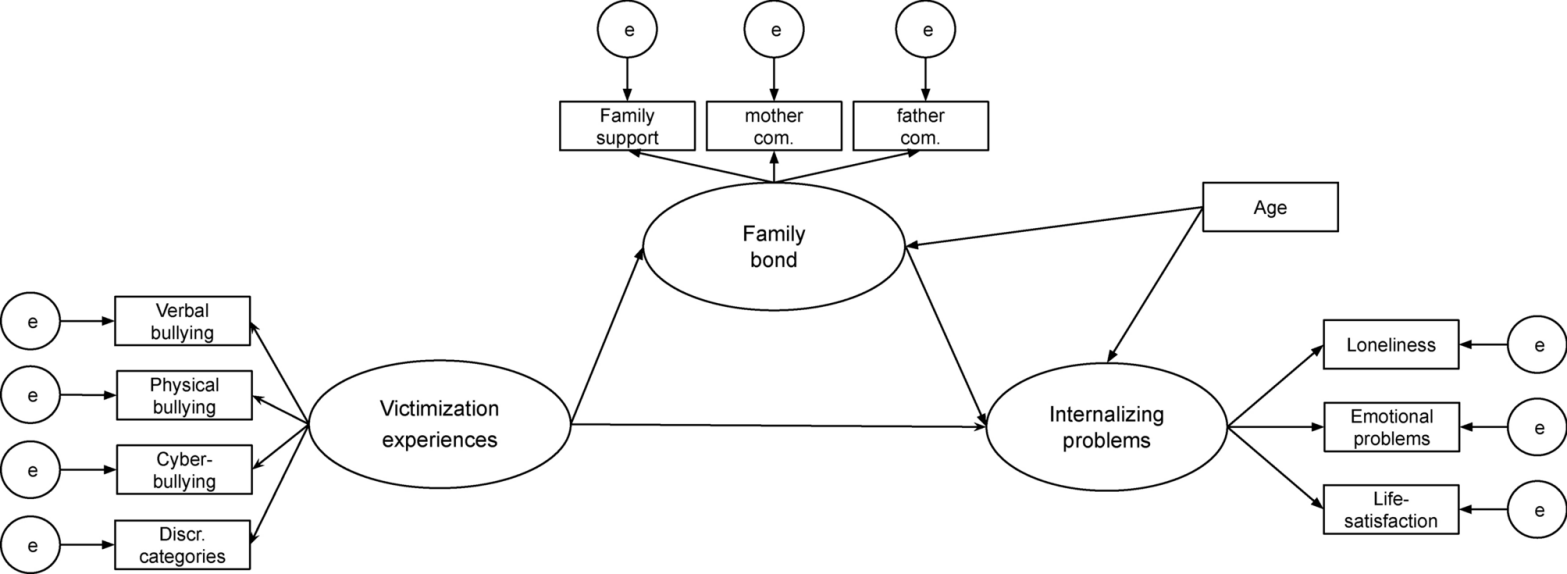
Conceptual model.

A multigroup SEM was then performed to test the relationships between latent variables across and within social groups, controlling for age (see Fig. 1 for the conceptual model). As monthly net household income was not significantly related to any relevant variable and did not differ significantly between the four social groups, it was not included as a control variable. The latent variable family bonds was then examined concerning the mediating influence between victimization experiences and internalizing experiences using the respective models. The multigroup structural equation models were conducted using a two-step approach^64^, which first validates the measurement model and then the overall model including the latent structures. To further explore the group differences in the path coefficients of relevant latent variables identified in the multi-group SEM, post-hoc pairwise comparisons were conducted using chi-square difference tests.

## Results

### Sample characteristics

After balancing the groups, the subsample used for the analyses comprised *n* = 1055 adolescents from the United Kingdom aged 10–15 years (*M* = 12.60 years, *SD* = 1.68; 52.7% female). The sample was divided into four social groups according to the participants’ gender (male, female) and self-identified ethnicity (White, BAME). The monthly mean net household income for the overall group was £4149.14 and did not differ between the groups. 83.3% of the sample lived in an urban area, with higher proportions amongst the BAME adolescents (male: 95.2%, female: 94.3%) compared to white adolescents (male: 73.9%, female: 72.6%).

### Descriptive statistics and bivariate correlations

Mean scores, standard deviation of all variables for the groups are summarized in Table 1, bivariate zero-order correlations among study variables are displayed in Table S1 (see Supplemental Materials). Both white male adolescents and BAME male adolescents reported the highest occurrence of physical bullying. White male adolescents reported the highest level of family support. White female adolescents reported the highest occurrence of verbal bullying and highest levels regarding the three observed internalizing variables (loneliness, emotional problems and life satisfaction). BAME female adolescents reported the highest number of different discrimination experiences, indicating a broad range of discriminatory experiences across multiple aspects of their identity. Further, they reported the highest scores for mother–child communication. Significant between-group mean differences were detected for all variables except cyberbullying and child- father communication.

**Table 1:** Means, standard deviations among observed variables.

	Total	White		BAME	
	*n* = 1055	Male	Female	Male	Female
		*n* = 280	*n* = 280	*n* = 219	*n* = 276
	M (*SD*)				
*Victimization experiences*					
Verbal bullying (V1)	1.42 (0.75)	1.44 (0.76)**	1.56 (0.85)**	1.36 (0.70)**	1.33 (0.66)**
Physical bullying (V2)	1.22 (0.55)	1.29 (0.63)***	1.20 (0.50)***	1.29 (0.65)***	1.13 (0.37)***
Cyberbullying (V3)	1.15 (0.45)	1.14 (0.45)	1.17 (0.47)	1.15 (0.45)	1.13 (0.42)
Discrimination categories (V4)	0.55 (1.07)	0.40 (0.87)***	0.66 (1.11)***	0.42 (0.96)***	0.69 (1.24)***
*Family bonds*					
Family support (F1)	2.73 (0.49)	2.84 (0.38)***	2.67 (0.54)***	2.77 (0.46)***	2.67 (0.52)***
Child- mother communication (F2)	3.90 (1.11)	3.73 (1.12)**	3.95 (1.09)**	3.86 (1.09)**	4.04 (1.11)**
Child-father communication (F3)	3.30 (1.24)	3.25 (1.21)	3.29 (1.23)	3.32 (1.21)	3.33 (1.30)
*Internalizing problems*					
Loneliness (I1)	1.48 (0.57)	1.41 (0.55)***	1.62 (0.59)***	1.37 (0.53)***	1.50 (0.56)***
Emotional Problems (I2)	3.39 (2.55)	2.99 (2.34)***	4.57 (2.74)***	2.42 (2.08)***	3.37 (2.44)***
Life satisfaction (I3)^a^	2.41 (1.28)	2.32 (1.18)***	2.67 (1.41)***	2.18 (1.17)***	2.43 (1.27)***

^a^Higher scores indicate less life satisfaction.

*= *p* < 0.05, ** = *p* < 0.01, *** = *p* < 0.001, conducted with Kruskal–Wallis tests.

### Confirmatory factor analysis and model specification

To specify a reference model, the measurement model was initially verified using a confirmatory factor analysis to estimate the values for the overall sample model. The reference model for the overall sample showed an excellent fit to the data (see Table 2), although scaled Chi^2^ value (*Chi*^*2*^(27) = 40.68, *p *=  0.044) was significant, which may reflect sensitivity to the large sample size^65^. Local fit was improved by re-specifying the model multiple times, including adding error covariances between items such as verbal and physical bullying, child-mother and child-father communication, as well as life satisfaction and family support. For the multi-group analysis, we first specified a configural model to test whether the basic structure of the model was consistent across the four groups. The fit indices for the configural model (Table 2) indicated that the model adequately described the data across groups. We then tested a constrained model to assess whether the relationships among variables were invariant across groups. The constrained model, which incorporated partial invariance, also showed an acceptable fit to the data, although the significant Chi^2^ value indicated some deviation, likely due to the large sample size^65^. Based on the recommended benchmark of ∆CFI < 0.01^63^, the model comparison showed that while some parameters could be constrained to be equal across groups without significantly worsening model fit, others could not, indicating differences in the relationships among variables across groups. Therefore, partial scalar invariance was provided by non-equal intercepts of emotional problems, life satisfaction, discrimination categories, verbal bullying and child-mother communication. Considering the unequal distribution of experiences of discrimination among female and BAME participants in comparison to male or white participants, as reflected in the descriptive data (see Table 1), the factor loading on discrimination categories on victimization experiences was freed. Given this partial invariance, physical bullying, cyberbullying, family support, child-father-communication and loneliness can be assumed to have the same underlying meaning across the groups, making the interpretation of group differences for these constructs valid. McDonald’s Omega range from 0.64 (victimization experiences among BAME females) to 0.82 (victimization experiences among white females). The factor loadings, reliabilities and *R*^*2*^ of the observed variables on the latent variables for each group are summarized in Table 3. Each group had different moderate to good factor loadings. The variance explanation of the latent variables by the observed variables had moderate to good effects and varied across the groups (see Table 3).

**Table 2:** Model fit statistics.

Models	Scaled χ^2^	df	CFI	RMSEA	SRMR	Δχ^2^	Δdf	ΔCFI	p-value
*CFA*									
Reference model	40.68	27	0.994	0.025	0.021				
Configural model	172.68	112	0.976	0.049	0.042				
Constrained model	217.79	133	0.964	0.055	0.057	65.68	21	0.090	< 0.001***
*Mediation SEM*									
Reference model	87.00	35	0.979	0.041	0.034				
Configural model	264.51	159	0.960	0.054	0.056				
Constrained model	310.84	180	0.948	0.057	0.066	46.33	21	0.03	0.002**

Δχ^2^ was calculated using standard χ^2^ estimators. Reference model is calculated for overall sample, *n* = 1055.

Scaled χ^2^ is estimated by Satorra and Bentler (2001) ^61^.

**Table 3:** Confirmatory factor analysis results per group.

	White						BAME					
	Male			Female			Male			Female		
Latent and observed variables	ω	Factor Loading	R^2^	ω	Factor Loading	R^2^	ω	Factor Loading	R^2^	ω	Factor Loading	R^2^
*Victimization experiences* Verbal bullying Physical bullying Cyberbullying Discr. categories	0.67	0.595*** 0.512*** 0.333*** 0.555***	0.45 0.30 0.44 0.29	0.82	0.790*** 0.535*** 0.428*** 0.870***	0.45 0.30 0.44 0.29	0.70	0.676*** 0.555*** 0.659*** 0.533***	0.44 0.21 0.28 0.40	0.64	0.663*** 0.456*** 0.533*** 0.637***	0.62 0.28 0.18 0.76
*Family bonds* Family support Mother communication Father communication	0.74	0.671*** 0.427*** 0.484***	0.73 0.09 0.13	0.73	0.785*** 0.451** 0.381***	0.73 0.09 0.13	0.74	0.865*** 0.289** 0.376***	0.58 0.31 0.28	0.75	0.715*** 0.543*** 0.461***	0.63 0.21 0.13
*Internalizing problems* Loneliness Emotional Problems Life Satisfaction	0.69	0.657*** 0.568*** 0.805***	0.50 0.47 0.41	0.76	0.684*** 0.680*** 0.856***	0.50 0.47 0.41	0.65	0.724*** 0.683*** 0.636***	0.39 0.40 0.72	0.73	0.623*** 0.629*** 0.858***	0.47 0.46 0.73

** = *p* < 0.01, *** = *p* < 0.001. Reliability measure using McDonald’s Omega. Values reported standardized.

### Multi group structural equation model

A multi-group structural equation model was then applied to examine the direct and indirect effect between victimization experiences, internalizing problems and family bonds for the social groups. The configural model demonstrated a good fit to the data (see Table 2) for the four groups, and the constrained model incorporating partial invariance showed an acceptable fit to the data with the aforementioned non-equal factor loading and intercepts. The bootstrapped parameters estimated for all four social groups are summarized in Table 4. Victimization experiences, family bonds and age explained between 46.2% (BAME male) to 81.8% (BAME female) of the variance in internalizing problems. Age and victimization experiences accounted for 9.6% of the variance in family bonds for BAME male adolescents up to 34.0% for white female adolescents.

**Table 4:** Mediating multigroup SEM results per social group.

	White						BAME					
	Male			Female			Male			Female		
Direct Effects	R^2^	Estimate (95% CI)	z (SE)	R^2^	Estimate (95% CI)	z (SE)	R^2^	Estimate (95% CI)	z (SE)	R^2^	Estimate (95% CI)	z (SE)
*Internalizing problems*	0.620			0.807			0.462			0.818		
Victimization experiences		0.516 [0.08, 1.08]	1.73 (0.30)		0.827 [0.29, 1.54]	− 0.22 (3.77)		0.698* [0.03, 1.31]	2.01 (0.35)		0.824 [0.24, 2.21]	0.05 (15.85)
*Family bonds*		− 0.855 [− 1.78, − 0.47]	− 1.40 (0.61)		− 1.16 [− 3.20, − 0.61]	− 0.11 (10.25)		− 0.410 [− 0.81, − 0.13]	− 0.11 (3.84)		− 1.416 [− 3.23, − 0.75]	− 0.07 (20.96)
Age		0.099 [− 0.08, 0.25]	1.17 (0.08)		0.211 [0.05, 0.52]	0.17 (1.21)		0.018 [− 0.12, 0.13]	0.15 (0.12)		0.097 [− 0.08, 0.35]	0.06 (10.61)
Family bonds	0.191			0.340			0.096			0.216		
Victimization experiences		− 0.397* [− 0.71, − 0.10]	− 2.59 (0.15)		− 0.716*** [− 1.06, − 0.49]	− 4.87 (0.15)		− 0.326* [− 0.69, − 0.07]	2.07 (0.16)		− 0.492** [− 0.86, − 0.25]	− 3.15 (0.16)
Age		− 0.164** [− 0.01, 0.49]	− 2.68 (0.06)		0.024 [− 0.08, 0.11]	0.48 (0.05)		0.009 [− 0.09, 0.12]	0.17 (0.05)		− 0.107* [− 0.21, − 0.01]	− 2.09 (0.05)
*Indirect effects on internalizing problems*												
VE → FB → IP		0.340 [0.11, 0.91]	1.28 (0.26)		0.830 [0.04, 2.40]	0.08 (9.77)		0.134 [0.02, 0.39]	0.03 (4.63)		0.697 [0.03, 2.08]	0.08 (8.65)
Age → FB → IP		0.140 [0.03, 0.37]	0.98 (0.14)		− 0.211 [− 0.23, 0.11]	− 0.03 (0.88)		− 0.004 [− 0.07, 0.04]	− 0.02 (0.16)		0.152 [0.01, 0.52]	0.06 (2.50)

Visualization of the direct effects in the model can be seen in Fig. 1a, in the Supplement.

* = *p* < 0.05, ** = *p* < 0.01, *** = *p* < 0.001. Estimate reported unstandardized.

Regarding the first research question on the group differences on the relationship between victimization experiences and internalizing problems, results showed that higher levels of victimization experiences were significantly associated with increased internalizing problems solely among the group of male BAME adolescents (*b* = 0.698, *p* = 0.044). For all groups, no significant association was found regarding family bonds and internalizing problems. Victimization experiences were negatively associated with family bonds across all groups; however, the magnitude of this association differed among the groups. The strongest negative association was observed in white females (*b* = 0.716, *p *< 0.001), followed by BAME females (*b *= − 0.492, *p *< 0.01), white males (*b *= − 0.397, *p* = 0.013), and BAME males (*b *= − 0.326, *p *= 0.042). Post-hoc chi-square difference tests revealed no statistically significant differences in the path coefficients between any of the groups (e.g., white females vs. BAME males: *Chi*^*2*^(77) = 161.63, *p *> 0.05)

Furthermore, age exhibited a negative relationship with family bonds, with a more pronounced effect observed in white male adolescents (*b* = − 0.164, *p* = 0.007) compared to BAME female adolescents (*b* = − 0.107, *p* = 0.036). No significant association between age and family bonds was identified in the other groups.

Concerning the exploratory hypothesis on the mediating impact of family bonds on the influence of victimization experiences on internalizing problems, there were no significant indirect effects across all groups. Given the absence of indirect effects, the main effects can be interpreted as total effects.

## Discussion

This study examined group differences in the strength of the relationship between victimization experiences, such as discrimination and bullying, internalizing problems, and the influence of family bonds from an intersectional perspective. Regarding the first research question and the hypothesized positive association between victimization experiences and internalizing problems, only the BAME male adolescent group showed a statistically significant association. Contrary to our hypothesis, the BAME female group did not exhibit the strongest association between victimization experiences and internalizing problems compared to the other groups. This association was only evident among BAME male adolescents in our sample. Future replication studies and longitudinal analyses will provide further insights into the causal directions. Based on this, Bernard, Smith, and Lanier (2022) found a positive relationship between racial discrimination and internalizing problems among BAME adolescents, though their study did not find gender differences^66^. However, our data contradicted our assumption for BAME female participants and is therefore inconsistent with previous literature, which highlighted an equal^67,68^ or stronger effect^51,69^ of (racial) bullying on internalizing disorders for females. This discrepancy between previous studies and our results might be due to differences in how victimization experiences were operationalized across studies. In this study, self-reported experiences of discrimination, verbal, physical, and cyberbullying were considered as victimization experiences, whereas other studies may have excluded (racial) discrimination but included sexual harassment as a component of bullying^51^, potentially leading to different gender effects. Additionally, male adolescents may be more likely to report bullying and discrimination than females, according to previous studies^68,70^, potentially masking an association with internalizing problems among the female participants. A further consideration for the partly contradictory findings might relate to the increased prevalence of physical bullying among the male participants seen in our data and reported by other studies^69^. Physical violence has been associated with increased internalizing symptoms^71^ and lower mental health compared to racial discrimination experiences^72^ among BAME adolescents. Additionally, gender-specific differences in ethnic-racial socialization might account for these findings. Ethnic-racial socialization refers to how and whether parents thematize their ethnic group affiliation, educate their children about heritage, and prepare them for potential discrimination^73^. Supporting this, studies reported less racial and ethnic socialization among males than females^74^, which might lead to different coping strategies for (racial) bullying or discrimination^75,76^. For example, higher levels of ethnic-racial socialization were associated with a greater use of proactive coping strategies for dealing with discrimination experiences^77^. Racial socialization and gender-specific coping strategies are potentially important influencing factors for the relationship between victimization and internalizing symptoms and are promising constructs to be included in future studies.

Contrary to our hypothesis, no significant relationship was found between family bonds and internalizing problems in any of the groups. This finding contrasts with previous research that has underscored the protective role of family bonds in reducing internalizing problems, particularly among African American^78–81^ and female adolescents^82,83^. A potential explanation for this discrepancy could be the self-reported and cross-sectional design of our study, which might not fully capture the dynamic and situational variability of family interactions, especially during adolescence^84^. Future research could benefit from utilizing a diary study approach to capture these dynamic interactions more comprehensively.

Across all groups, a negative relationship was found between victimization experiences and family bonds, indicating that as victimization experiences increased, participants reported lower levels of family bonds. This finding aligns with a systematic review^85^, that reported 70% of included studies demonstrated a negative relationship between bullying victimization and parental support and involvement. However, post-hoc tests revealed no statistically significant differences between any of the groups, meaning that while within groups, significant relationships between victimization experience and family bonds were observed, these relationships did not significantly differ across groups. Additionally, a negative association concerning family bonds and age was observed among white male and BAME female participants, with higher age associated with lower levels of family bonds. This result may be linked to an increasing detachment from the family or parents and a shift towards an individual identity^84,86^.

Furthermore, we did not find a mediating effect of family bonds regarding the relation between victimization experiences and internalizing problems. This diverges from previous studies that have suggested a protective role of family^51,87^. In the complex association between victimization experiences and mental health outcomes, factors beyond family bonds, such as coping strategies^88^, racial socialization, parental worries, and parental mental health^89^, may also play a significant role. Since this information was not collected in our study, future research analyzing this data is recommended.

In summary, the hypothesized negative association between victimization experiences and internalizing symptoms was observed only among BAME male adolescents. Additionally, while family bonds were negatively associated with victimization experiences across all groups, no significant differences were found between the groups. However, the significance of the relationship between victimization experiences and internalizing problems solely for BAME males underscores the importance of an intersectional approach. Such an approach is essential for understanding the unique and compounded effects of victimization on mental health among adolescents.

## Limitation

This study is a cross-sectional analysis, impeding conclusions about longitudinal effects. Although the McDonald’s omega group values for victimization experiences of 0.64–0.82 are close to the acceptable value of 0.70 for studies^90^, it should be noted that the items used in the Panel Study for assessing bullying and discrimination categories had not been validated. This must be taken into account when considering the replication of the findings. Moreover, the data collection period encompassed phases of the COVID-19 pandemic lockdown, a factor that might contribute to psychological strain^91^, family bonds^92^ and victimization experiences^93^ and that might limit the comparability of findings with data from pre-pandemic times. Class, as a key dimension of marginalization and intersectionality, has been shown to influence the relationship between bullying victimization and internalizing symptoms^94^. However, in our sample, socio-economic status was not related to any relevant variables and did not differ between the groups. Taking a closer look at the descriptive data on net income within our sample, the income is to be classified as a slightly above-average to middle income, compared with the data of the Office of National Statistics in the UK^95^. Since postal recruitment is related to socio-economic factors such as a registered residential address, a possible consequence could be a biased sample towards middle-income participants and bypass potential low-income or poverty-affected groups^96^. Therefore, a sample with diverse socioeconomic backgrounds is essential for ensuring the generalizability of findings in future studies. Also, in the framework of a quantitative study design, it should be noted that an adequate depiction of individual life realities and the incorporation of multiple intersectionalities is challenging, supporting the urgent demand for validated measurement instruments for these domains for adolescents.

However, this study’s intersectional approach was fundamental to our understanding of victimization experiences, allowing us to capture the unique and compounded effects of race and gender on internalizing problems. The findings underscore the importance of recognizing these intersecting dynamics when examining the psychological impact of victimization and therefore contributed considerably to bridging a research gap regarding an intersectional approach towards victimization experiences and internalizing problems in adolescents. Future studies should put a more specific focus towards systemic protective factors and coping factors concerning victimization experiences. Early parental influence factors like ethnic-racial socialization should receive more attention when it comes to research questions among adolescents. Lastly, a longitudinal study design will provide insights on temporal relationships between the variables of interest and the assumed mediational effects of family effects on experiences of victimization and should therefore be aimed for in a future design.

## Conclusion

In conclusion, our findings underscore the critical need for an intersectional approach in research. Moving beyond singular or multi-sectional perspectives, integrating the complex interplay of social groups and related discrimination categories such as gender and race is essential. By recognizing and incorporating the lived realities of individuals experiencing discrimination, particularly in childhood and adolescence, research and clinical practice can better address the multifaceted nature of mental health problems in this crucial developmental stage. Moreover, this study highlights the importance of tailored interventions for BAME male adolescents, with a specific focus on addressing potential victimization experiences, which can be done both within school settings and psychotherapeutic or counseling contexts. Preventive approaches such as anti-bullying programs should incorporate strategies that address physical violence and its severe psychological impact, with a focus on BAME communities. Furthermore, school-based mental health services should be equipped to support adolescents who experience racial victimization, offering tailored interventions that consider the intersectionality of these experiences. Therefore, policies should be designed to ensure that anti-bullying initiatives are inclusive of race and gender considerations, providing specific guidelines for how schools should address these overlapping forms of victimization. While family bonds may not directly mediate the impact of victimization on internalizing problems, the significant association between family bonds and internalizing problems underscores the relevance of family-centered interventions in clinical care. By prioritizing intersectionally-informed approaches, we can strive towards more equitable and effective strategies for addressing the mental health needs of vulnerable populations.

## Supplementary Information

Supplementary Information.

## Data Availability

The initial Understanding Society datasets generated and analysed during the current study are available in the UK Data Service repository, https://beta.ukdataservice.ac.uk/datacatalogue/series/series?id = 2,000,053. However, the cleaned dataset used and analysed during the current study available from the corresponding author on reasonable request.
