# Supplementary material for: Victimization experiences, internalizing problems and family bonds among adolescents from the UK: multi-group structural equation modeling using an intersectionality-informed approach
Source: Sci Rep. 2024 Nov 20;14:28753. doi: 10.1038/s41598-024-80342-0 (PMC11579348; doi:10.1038/s41598-024-80342-0)
Supplement: Supplementary file 1 — Supplementary Information. [file 41598_2024_80342_MOESM1_ESM.docx]

**Supplemental Material**

S1

| **Variable** | 1 | 2 | 3 | 4 | 5 | 6 | 7 | 8 | 9 | 10 |
| --- | --- | --- | --- | --- | --- | --- | --- | --- | --- | --- |
| 1. Verbal Bullying | 1 | .53**  /.52** | .40**  /.41** | .69**  /.40** | -.29**  /-.21** | -.24**  /-.07 | -.17**  /-.12* | .41**  /.32** | .43**  /.33** | .45**  /.33** |
| 2. Physical Bullying | .61**  /.74** | 1 | .23**  /.21** | .48**  /.31** | -.26**  /-.15* | -.13*  /-.04 | -.12*  /-.04 | .26**  /.20** | .24**  /.29** | .30**  /.21** |
| 3. Cyberbullying | .23**  /.43** | .26**  /.35** | 1 | .32**  /.33** | -.23**  /-.19** | -.09  /-.17** | -.08  /-.13* | .30**  /.18** | .30**  /.24** | .29**  /.25** |
| 4. Discrimination categories | .33**  /.33** | .27**  /.27** | .10  /.41** | 1 | -.43**  /-.24** | -.25**  /-.10 | -.11  /-.13* | .42**  /.33** | .50**  /.26** | .54**  /.39** |
| 5. Family support | -.09  /-.18* | -.14**  /-.12 | -.24**  /-.21** | .12*  /-.16* | 1 | .36**  /.39** | .29**  /.33** | .44**  /.42** | .44**  /.35** | .58**  /.54** |
| 6. Child-Mother communication | -.03  /.07 | -.02  /.12 | -.05  /-.01 | -.09  /.04 | .26**  /.26** | 1 | .56**  /.59** | .20**  /.21** | .12*  /.26** | .37**  /.42** |
| 7. Child-Father communication | -.21**  /-.04 | -.06  /-.02 | -.10  /-.03 | -.06  /.03 | .32**  /.33** | .56**  /.55** | 1 | .24**  /.25** | .13*  /.24** | .31**  /.33** |
| 8. Loneliness | .24**  /.34** | .17**  /.33** | .15*  /.27** | .31**  /.21** | .28**  /.28** | .21**  /.02 | .16**  /.14* | 1 | .60**  /.51** | .59**  /.52** |
| 9. Emotional Problems | .21**  /.34** | .13*  /.26** | .18**  /.27** | .27**  /.23** | .24**  /.20** | .06  /.04 | .11  /.07 | .56**  /.40** | 1 | .58**  /.55** |
| 10.Life satisfaction ^a^ | .19**  /.26** | .18**  /.20** | .12  /.15* | .24**  /.10 | .40**  /.36** | .31**  /.26** | .30**  /.30 | .52**  /.44** | .46**  /.46** | 1 |

**Table 2.** Zero - order correlations among observed variables among social groups. ^a^ Higher scores indicate less life satisfaction; * = *p* < .05, ** = *p* < .01. Correlations for female adolescents above the diagonal (White/BAME), for male adolescents reported below (White/BAME).

F1


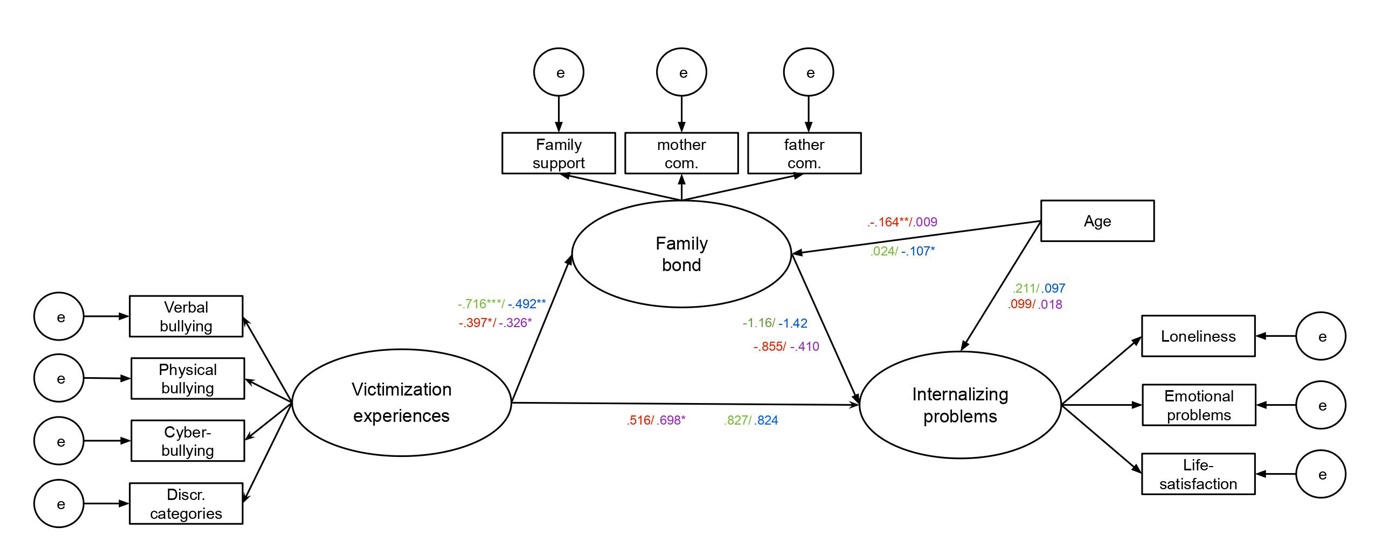


**Figu****re 1a.** Conceptual model with unstandardized direct effect estimates. Coefficients for female adolescents (White/BAME), for male adolescents (White/BAME) are indicated with distinct colors. * = *p* < .05, ** = *p* < .01, *** = *p* < .001.
